# Spatiotemporal Thermal Variations in Moroccan Cities: A Comparative Analysis

**DOI:** 10.3390/s23136229

**Published:** 2023-07-07

**Authors:** Ahmed Derdouri, Yuji Murayama, Takehiro Morimoto

**Affiliations:** Faculty of Life and Environmental Sciences, University of Tsukuba, 1-1-1 Tennodai, Tsukuba 305-8572, Japan; mura@geoenv.tsukuba.ac.jp (Y.M.); tmrmt@geoenv.tsukuba.ac.jp (T.M.)

**Keywords:** Land Surface Temperature (LST), Moroccan urban landscapes, machine learning

## Abstract

This study examines the Land Surface Temperature (LST) trends in eight key Moroccan cities from 1990 to 2020, emphasizing the influential factors and disparities between coastal and inland areas. Geographically weighted regression (GWR), machine learning (ML) algorithms, namely XGBoost and LightGBM, and SHapley Additive exPlanations (SHAP) methods are utilized. The study observes that urban areas are often cooler due to the presence of urban heat sinks (UHSs), more noticeably in coastal cities. However, LST is seen to increase across all cities due to urbanization and the degradation of vegetation cover. The increase in LST is more pronounced in inland cities surrounded by barren landscapes. Interestingly, XGBoost frequently outperforms LightGBM in the analyses. ML models and SHAP demonstrate efficacy in deciphering urban heat dynamics despite data quality and model tuning challenges. The study’s results highlight the crucial role of ongoing urbanization, topography, and the existence of water bodies and vegetation in driving LST dynamics. These findings underscore the importance of sustainable urban planning and vegetation cover in mitigating urban heat, thus having significant policy implications. Despite its contributions, this study acknowledges certain limitations, primarily the use of data from only four discrete years, thereby overlooking inter-annual, seasonal, and diurnal variations in LST dynamics.

## 1. Introduction

Land Surface Temperature (LST) is a critical parameter in the fields of climatology, hydrology, and environmental science, playing a significant role in the energy balance of the Earth’s surface [1]. It is influenced by a variety of factors, including vegetation cover, surface water, and terrain characteristics, and can have significant impacts on local and regional climate patterns [2].

Globally, the study of LST has received significant attention, with a proliferation of studies investigating the factors influencing LST across various geographical settings and utilizing various analytical techniques. A global review of such studies reveals diverse factors affecting LST, which include but are not limited to land use/land cover (LULC), altitude, population density, vegetation index, albedo, and surface moisture [3]. Many studies have also highlighted the growing impact of anthropogenic activities, particularly urbanization and deforestation, on LST [4,5]. This is evident in the increasing incidence of the urban heat island (UHI) effect, a phenomenon where urban regions exhibit higher temperatures than surrounding rural areas, as documented in studies across various continents [6].

Satellite remote sensing, particularly MODIS (Moderate Resolution Imaging Spectro-radiometer) and Landsat platforms, has been instrumental in investigating LST at varying spatial and temporal resolutions. These tools provide opportunities to monitor LST dynamics over large areas, supplementing in situ observations that often suffer from limitations related to coverage and continuity. While both MODIS and Landsat have contributed immensely to LST studies, there has been a more substantial volume of research conducted using MODIS due to its higher temporal resolution, facilitating more frequent LST measurements [3,6]. Notably, MODIS provides near-daily global coverage, which is particularly advantageous in capturing short-term thermal variations. Landsat, on the other hand, is known for its finer spatial resolution, contributing to detailed investigations at local scales.

Research methodologies have evolved over the years, with a noticeable shift from traditional statistical methods to more advanced analytical techniques like machine learning (ML). The use of ML in LST research has proved beneficial in understanding complex patterns and relationships that traditional statistical methods may overlook [3]. The integration of satellite-based remote sensing data with ML models has provided a powerful tool for investigating LST on a global scale, allowing researchers to conduct more accurate and efficient studies [6]. ML algorithms such as eXtreme Gradient Boosting (XGBoost) and LightGBM, which are used in this study, provide ways to handle complex and non-linear relationships between variables, capture spatial autocorrelation, and address issues of heteroscedasticity and multicollinearity commonly encountered in LST research. Despite their benefits, few studies have leveraged these methods in understanding LST, especially in the context of Moroccan cities.

To address these gaps, our study incorporates advanced ML models, including XGBoost and LightGBM, and applies a rigorous cross-validation and performance evaluation framework. The XGBoost and LightGBM models, known for their high performance and computational efficiency, offer a robust approach to handling the complex, multi-dimensional nature of LST datasets. These models allow for the handling of non-linear relationships, accommodate interactions between variables, and have inbuilt mechanisms for avoiding overfitting. We complement these models with SHAP (SHapley Additive exPlanations) values, providing an interpretable measure of feature importance and thus offering insights into the key drivers of LST. By addressing these limitations in previous studies, we aim to develop a more accurate, robust, and interpretable understanding of LST dynamics in Moroccan urban environments.

In the context of Moroccan cities, understanding LST is particularly important due to the country’s diverse geographical features, ranging from coastal areas to inland regions with varying topography. Morocco’s unique geographical position, straddling both the Mediterranean and Atlantic coasts and being home to parts of the Sahara desert, makes it a compelling case study for LST analysis. Moreover, rapid urbanization and land use changes in Moroccan cities over the past few decades [7] have likely had significant impacts on LST, making this a timely and relevant area of study.

Several studies have been conducted to investigate LST and the UHI phenomenon in Moroccan cities (Table 1), focusing on factors such as vegetation, built-up areas, and land cover. Rhinane et al.’s 2012 study used Landsat 5 TM images to investigate Casablanca’s ground temperature. They found that vegetation significantly correlated with cooler areas, emphasizing its role in reducing UHIs [8]. Lachir et al. (2016) combined Landsat and MODIS data to evaluate the urbanization effects on Marrakech’s surface climate. They found significant variations in the growing season surface temperature differences between urban and other cover types, highlighting the influence of urbanization on surface climate [9]. Bahi et al. (2016) utilized a sequence of Landsat TM/ETM+/OLI-TIRS images to track the spatial dispersal of Surface UHI (SUHI) in Casablanca. Their findings indicated a unique seasonal cycle of daytime SUHI in Casablanca, differing from other mid-latitude cities, which highlights the necessity for localized research [10]. In a comprehensive study on UHIs in Morocco, Fathi et al. (2019) discovered a pronounced UHI effect in urban regions established within green lands. Conversely, they identified an urban heat sink (UHS) phenomenon in cities built in arid zones [11]. El Ghazouani et al. (2021) used Landsat-8 surface temperature and the European Space Agency land cover data to assess the impact of land cover on the UHI and UHS in five Moroccan cities. They found multiple causes defining the different forms and amplitudes of the UHI [12]. Lastly, Gourfi et al. (2022) used air temperature measurements and spatial analysis to investigate the interplay among green spaces, constructed regions, and SUHI in Marrakesh. Their investigation revealed a mean LST variance of 3.98 °C across diverse city neighborhoods [13].

While these studies offer valuable insights into the UHI phenomenon in Moroccan cities, several gaps remain unaddressed. Notably, earlier studies did not consider relevant factors such as terrain characteristics and did not utilize advanced analytical methods such as ML. Furthermore, most of the studies focused on individual cities and did not provide a comprehensive analysis of multiple cities over an extended period. These gaps present opportunities for further research, such as the current study, which aims to analyze the contributing factors of LST in eight Moroccan cities over a 30-year period using spectral indices, terrain characteristics, and ML methods.

Responding to these limitations, our research seeks to bridge these knowledge gaps and contributes a novel approach to understanding the dynamics of LST in Moroccan urban environments. The primary objectives of our study are as follows:

- To perform a comprehensive analysis of LST trends across eight Moroccan cities over the past three decades, thereby extending the temporal scope of existing research.
- To identify and analyze the impact of spectral indices and terrain characteristics on LST in the selected cities between 1990 and 2020, thus broadening the range of contributing factors considered in such studies.
- To compare the thermal environments of coastal and inland cities to uncover any distinctive patterns or differences.

By addressing these questions, this study aims to contribute to the understanding of LST dynamics in Moroccan cities and provide a foundation for future research in this area, which is currently limited in terms of studies addressing such research topics [3].

The remainder of this paper is organized as follows: Section 2 provides a comprehensive description of the target cities, the data collection and processing procedures, the spectral indices and terrain characteristics used, and the employed methods including Geographically Weighted Regression (GWR) and ML models (XGBoost and LightGBM) along with the use of SHAP values. Section 3 presents the findings from the visual analysis of spatiotemporal LST trends, the ML models’ performance assessment, and the interpretation of their output using the SHAP values. Section 4 interprets the results in the context of the research objectives, compares the results with previous studies, discusses the implications, clarifies the limitations, and suggests future directions. Finally, Section 5 provides the concluding remarks.

## 2. Materials and Methods

### 2.1. Target Cities

**Figure 1:**
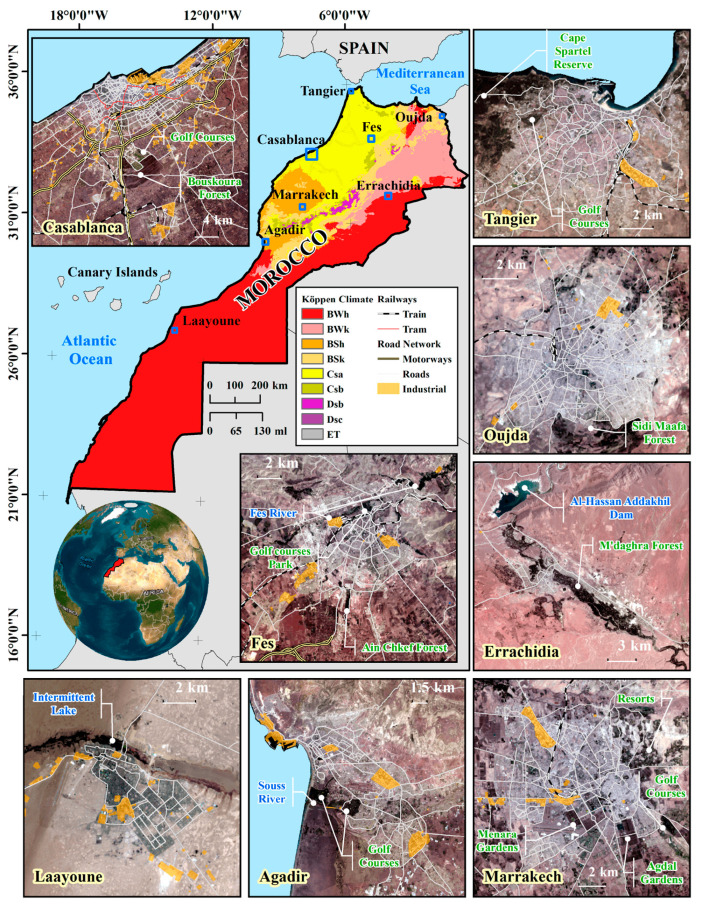
Map of Morocco Köppen-Geiger climate classification [14] highlighting target cities and their geographical settings: Casablanca, Tangier, Agadir, Fes, Marrakech, Oujda, Laayoune, Errachidia. Landsat 8 and 9 images produced by the U.S. Geological Survey. Industrial zones and transportation networks data © OpenStreetMap contributors [15].

The focus of this study encompasses eight cities within Morocco (Figure 1 and Table 2), each chosen to encapsulate a broad spectrum of geographical attributes and climatic zones. These cities, a mix of coastal and inland regions, exhibit diverse topography, land use patterns, and population densities, thereby providing a comprehensive representation of the different environments present in Morocco.

Casablanca is situated along the Atlantic coast within the Mediterranean climate zone [14]. As of 2014, it hosts a population of 3.4 million [16], making it the third-most major populous agglomeration in North Africa, following Cairo and Alexandria in Egypt [17]. As the economic epicenter of Morocco, it serves as the domicile for numerous multinational corporations, establishing its significance on a global economic scale. The built-up area of Casablanca expanded by 38.32% from 2000 to 2015 and saw further growth of 63.54% between 1984 and 2018, with urban development especially notable towards the south in the direction of Bouskoura Forest, a prominent green area adjacent to the city [7,18].

Tangier, boasting a population of around 1 million as of 2014 [16], ranks as Morocco’s second-largest economic hub [19]. Located strategically on the Strait of Gibraltar, this coastal city stands at an elevation of 21 m. The city’s vast and dynamic expansion, notably influenced by the development of Tangier-Med—a significant cruise and cargo port [19]—is marked by a 43.71% increase in its built-up area from 2000 to 2015 [7]. The city, part of the larger Tangier-Tetouan-Al Hoceima region, lies within the hot-summer Mediterranean climate zone [14]. This region experiences diverse climatic conditions due to its geomorphological configuration, which combines the high Rif mountains and coastal plains, leading to varied microclimates [20].

Agadir, a coastal city situated in the Souss-Massa region of Morocco, is nestled within the hot semi-arid climate zone [14]. As of 2014, it is home to a population of 600,599, making it one of the major urban centers in the country [16]. The city’s economy, primarily driven by tourism, agriculture, and fishing, contributes significantly to the national GDP. Agadir’s built-up area expanded by 66.86% between 1986 and 2019 [21], reflecting its rapid urban development. Agadir’s unique geographical location, with the Atlantic Ocean to its west and the Anti-Atlas mountains to its east, creates a distinctive blend of coastal and mountainous landscapes leading to notable climate variations, adding to the city’s environmental diversity and complexity.

Fes, situated at an elevation of 409 m, is an inland city with a population of approximately 1.2 million [16]. The city is exposed to a hot-summer Mediterranean climate [14]. From 2000 to 2015, the city expanded by 24.32%, reflecting significant urban development [7]. Fes el Bali, the city’s historic medina and a UNESCO World Heritage site, is distinguished by its pedestrian-centric urban layout and traditional mud and adobe architecture, which serve dual roles of aligning with Moroccan architectural aesthetics and cultural traditions while also regulating indoor temperatures due to their high thermal mass [22,23].

Marrakech, known as the “Red City” for its distinctive red sandstone buildings, is an inland city with a population of 1.4 million [16]. The city, located at an elevation of 466 m, experiences a hot semi-arid climate [14]. It saw a significant urban expansion of 62.33% from 2000 to 2015 [7]—marking one of the highest percentages of urban growth among Moroccan cities during this period. Its historic medina, a UNESCO World Heritage site, sits alongside modern urban spaces and well-known gardens like the Agdal and Menara.

Oujda, an inland city situated in northeastern Morocco, is home to a population of approximately 506,274 people [16]. The city experiences a unique blend of climates, characterized by a mix of semi-arid and desert climates [14]. Geographically, Oujda is located at an elevation of 540 m above sea level. From 2000 to 2015, the city underwent a significant transformation in terms of its built-up areas, with a notable increase of 36% in built-up change [7].

Laayoune, a near-coastal city in the desert climate zone [14], has a population of 238,096 [16]. Located at an elevation of 64 m, Laayoune is the largest city in southern Morocco and serves as a hub for phosphate mining, one of the region’s primary industries.

Errachidia, an oasis city with a population of 418,451 [16], is recognized for its combined hot and cold desert climate [14]. The city resides at a 972 m elevation and is known for its abundant sunshine, averaging 330 days a year [24]. Its ecological significance was highlighted when its oases were declared a UNESCO biosphere reserve in 2000. The city experienced a high urbanization rate of more than 40% between 2004 and 2014 [24].

The selection of these cities, each with its unique geographical and climatic characteristics, allows for a robust analysis of the factors contributing to LST across different Moroccan cities. This study aims to leverage the diversity of these cities to gain a comprehensive understanding of the factors influencing LST in Morocco. Table 2 provides a summary of the key characteristics of each city.

### 2.2. Methods

**Figure 2:**
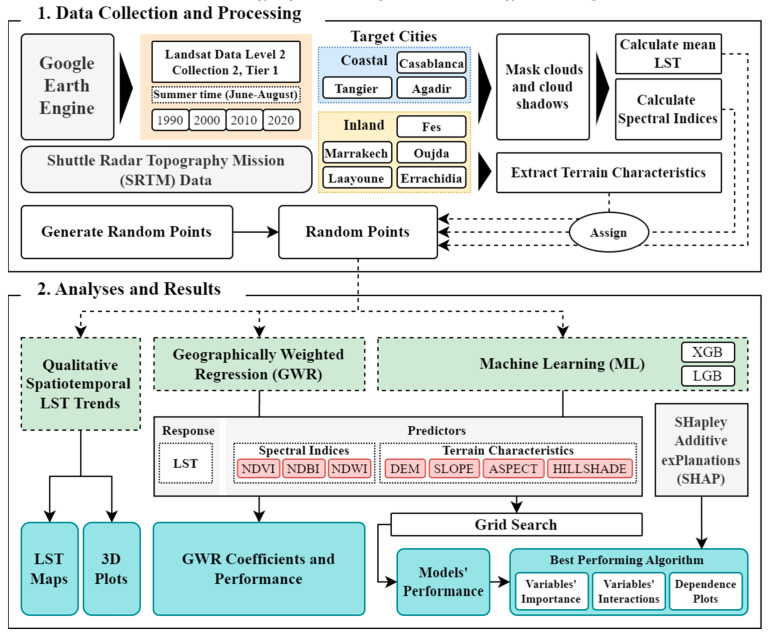
Methodology flowchart.

Figure 2 illustrates the methodological process of our research. The flowchart commences with data collection and processing (Section 2.2.1), followed by a series of analyses to derive the results. Initially, we gathered Landsat and Shuttle Radar Topography Mission (SRTM) data [25], processed them to calculate LST and spectral indices, and assigned these values to 3000 random points within the extent of selected Moroccan cities (Section 2.2.2). Next, a qualitative analysis of spatiotemporal LST trends was conducted, followed by a GWR analysis to ascertain relationships between LST, spectral indices, and terrain characteristics (Section 2.2.3). Finally, we used ML algorithms, namely XGBoost and LightGBM to delve deeper into the relationship between LST and the predictors (Section 2.2.4). A grid search procedure helped identify the best hyperparameters, and the top-performing model was chosen for further examination. These models were interpreted using SHAP values to understand the influence of various factors on predicted LST.

#### 2.2.1. Data Collection and Processing

The data collection and processing for this study were conducted using Google Earth Engine (GEE), a cloud-based platform for planetary-scale geospatial analysis [26]. The data were sourced from Landsats 5 and 8 (Level 2, Collection 2, Tier 1), which have already undergone geometric and atmospheric correction. These satellite images were specifically chosen from the summer months, a period when the UHI effect is more pronounced.

For each city and year, the images were first cleaned of cloud pixels. Following this, the images were stacked, and a mean image was extracted. This mean image was then masked based on the study area extent, which was carefully selected to encompass the latest extent of urban areas for each city. It is important to note that data for Agadir in 1990 were not available, and as such, data from 1995 were used as a suitable alternative.

In addition to the Landsat data, Digital Elevation Model (DEM) data were obtained from the SRTM data. The DEM data provided valuable information about the topography of the study areas, which was crucial in understanding the natural characteristics influencing the LST. Subsequent processing of the DEM data using Geographic Information System (GIS) software allowed for the extraction of additional characteristics such as slope, hillshade, and aspect. Terrain characteristics significantly influence LST. For instance, a study conducted in Hangzhou, China, found that elevation and slope are negatively correlated with LST, with higher altitudes and steeper slopes having lower LST [27]. Shaded relief (hillshade) also impacts LST, with more shadows leading to lower LST [27]. Aspect has a less significant relationship with LST, with higher values on southern-facing slopes [27]. Further, a study on Kilimanjaro revealed that differences between LST and air temperature tend to increase with elevation [28]. In the semi-arid region of Abha-Khamis-Mushyet, LST was found to be significantly influenced by altitude and corresponding LULC types, with the highest LST in exposed rocky areas and built-up land and the lowest in dense vegetation [29]. Moreover, a study in Cameron Highlands demonstrated that deforestation and urban development on slopes above 35° lead to soil structure instability and an increase in LST [30]. These findings highlight the importance of terrain characteristics in LST studies, particularly in comparative studies of cities with diverse topographic characteristics.

This comprehensive data collection and processing approach ensured a robust dataset, facilitating detailed analysis of the contributing factors to LST in the selected Moroccan cities. The use of GEE and GIS software allowed for the efficient and accurate processing of large volumes of satellite and topographic data, ensuring the reliability and validity of the study’s findings.

#### 2.2.2. Extraction of Land Surface Temperature (LST) and Spectral Indices

LST is a critical parameter in the physics of land surface processes on a global scale, directly linked to the energy balance at the Earth’s surface. It is a measure of the heat radiated by the land surface and can be influenced by various factors such as vegetation cover, soil moisture, and urban materials. In this study, LST was directly derived from the surface temperature bands of the Landsat 5 (ST_B6) and Landsat 8 (ST_B10) using the Python package arcpy. Next, we applied the corresponding scale factors and constants for each Landsat version to convert the output to Kelvin, followed by a subtraction of 273.15 to convert Kelvin to degrees Celsius.

In terms of estimated errors in the LST product, factors such as sensor calibration, radiometric noise, and atmospheric interference could cause slight deviations. However, these errors are expected to be low because the Collection 2, Tier 1 data from Landsat have undergone stringent radiometric calibration and atmospheric correction processes. Despite this, it is generally accepted that the LST derived from Landsat data can have an uncertainty of approximately ±1–2 °C under optimal conditions. This uncertainty has likely been reduced in our analysis due to the use of mean values from multiple cloud-free images.

In addition to LST, spectral indices were also extracted from the Landsat datasets. These indices, namely the Normalized Difference Vegetation Index (NDVI), Normalized Difference Water Index (NDWI), and Normalized Difference Built-up Index (NDBI), are key proxies for analyzing land cover types of the study areas. The selection of these specific indices is grounded in their proven effectiveness and widespread use in LST studies [3,6].

NDVI is a widely used index for assessing vegetation cover and health [31]. It is calculated using the near-infrared (NIR) and Red bands of the Landsat data (Equation (1)). Higher NDVI values are indicative of denser and healthier vegetation.

NDVI = (NIR − Red)/(NIR + Red)(1)

NDWI is used to monitor changes in water content in vegetation and can also be used to detect open water surfaces [32,33]. It is calculated using the Green and NIR bands of the Landsat data (Equation (2)), with higher values indicating higher water content.

NDWI = (Green − NIR)/(Green + NIR)(2)

NDBI is used to identify built-up areas [34]. It is calculated using the short-wave infrared (SWIR) and NIR bands of the Landsat data (Equation (3)), with higher values indicating denser built-up areas.

NDBI = (SWIR − NIR)/(SWIR + NIR)(3)

#### 2.2.3. Geographically Weighted Regression (GWR) Analysis

GWR is a local version of spatial regression that generates parameters disaggregated by the spatial units of analysis. This technique allows for the identification of relationships and patterns that can vary spatially, providing a more nuanced understanding of the studied phenomena. GWR is particularly useful in the context of urban studies, where relationships between variables can be expected to change across the urban landscape due to the spatial heterogeneity inherent in urban areas [35].

GWR is particularly useful in LST analysis, as it can account for the spatial heterogeneity inherent in environmental data and provide localized parameter estimates that can reveal complex spatial patterns and relationships. Previous studies have successfully applied GWR to analyze the spatial patterns of LST and its impact factors. For instance, Zhao et al. (2018) used GWR to explore the spatial non-stationarity and scale effects of the relationships between LST and related impact factors at multiple resolutions in Zhengzhou City, China [36]. Similarly, Zhi et al. (2020) employed GWR to analyze the driving factors and spatial heterogeneity of LST in the Xigang District of Dalian City, China [37]. Lu et al. (2021) utilized GWR to assess the impact of LST on urban net primary productivity increments [38].

These studies demonstrate the utility of GWR in understanding the spatially varying relationships between LST and various factors. In this study, GWR was employed to investigate the relationship between LST and the selected factors, namely the spectral indices (NDVI, NDWI, NDBI) and natural characteristics (DEM, SLOPE, ASPECT, HILLSHADE). This approach allowed for a nuanced understanding of how these factors contribute to LST in different geographical contexts within the selected Moroccan cities.

#### 2.2.4. Machine Learning (ML) Analysis of LST and Contributing Factors

ML has surfaced as a potent tool for parsing complex environmental data, revealing intricate interdependencies between variables. This study employed ML algorithms, specifically XGBoost and LightGBM, to delineate the relationship between LST and selected factors including spectral indices (NDVI, NDWI, NDBI) and natural characteristics (DEM, SLOPE, ASPECT, HILLSHADE).

XGBoost algorithm, an efficient realization of the gradient-boosting framework developed by Chen and Guestrin (2016) [39], is revered for its speed and performance. As an ensemble learning technique, XGBoost harnesses decision tree models to iteratively construct new models that predict residuals or errors of preceding models, culminating in a final prediction. This mechanism, dubbed “gradient boosting”, leverages a gradient descent algorithm to curtail the loss when adding new models [40]. Essential to this method is the strategic handling of missing data, accommodating categorical variables, and introducing mechanisms to manage model complexity via hyperparameters. This study explored hyperparameters like learning rate, max depth, min child weight, and others, instrumental in curbing model complexity and avoiding overfitting. We optimized these hyperparameters using a grid search strategy, ensuring a balance in the bias–variance trade-off.

Parallelly, LightGBM, another gradient-boosting framework developed by Microsoft, using tree-based learning algorithms, is architected to be both distributive and efficient [41]. It demonstrates superior training speed and efficiency, outperforming many competing algorithms when handling large datasets, attributable to novel techniques such as Gradient-based One-Side Sampling and Exclusive Feature Bundling. The LightGBM employs a leaf-wise growth strategy for tree growth, and like XGBoost, its hyperparameters—learning rate, number of leaves, regularization parameters, and others—control the model’s complexity and prevent overfitting. These were also fine-tuned using a grid search strategy.

The efficacy of these ML algorithms significantly depends on the setting of hyperparameters. To decipher the most potent combination, both models underwent a grid search strategy. The spectrum of hyperparameters considered in the grid search for both models is summarized in Appendix A, Table A1. The primary objective during the optimization of these hyperparameters was to identify the combination delivering the most accurate prediction on unseen data, achieved by minimizing the error between the model’s predictions and actual values.

The ML models were interpreted, and the contributions of each factor to the predicted LST were ascertained using SHAP values. SHAP values serve as a consolidated measure of feature importance and a potent tool for interpreting ML models [42]. These values provide insights into each feature’s contribution to the prediction for each observation, thus offering a nuanced understanding of the model’s behavior.

Alternative ML techniques, such as Support Vector Machines (SVMs), Random Forest (RF), and Artificial Neural Networks (ANNs), were evaluated during the conception of this study. Despite the potential of these algorithms, XGBoost and LightGBM were favored due to their unique capabilities in dealing with extensive datasets [40]. For instance, SVM, while potent, might not provide the same efficiency as our selected models when handling large datasets [43]. Similarly, while RF is a robust ensemble learning method, it does not offer the same depth of fine-tuning capabilities inherent in gradient-boosting techniques utilized by XGBoost and LightGBM [40,44]. The ANN, a complex and powerful tool, was also considered. However, despite its potential to capture non-linear relationships, ANN sometimes suffers from overfitting and interpretability issues [45], issues well-managed by XGBoost, LightGBM, and SHAP. Moreover, our choice of XGBoost and LightGBM was further justified by the compatibility of these algorithms with TreeExplainer. TreeExplainer is an implementation of Tree SHAP, a rapid and precise method used to compute SHAP values specifically for tree-based models and ensembles of trees. This unique alignment currently exclusively extends to XGBoost and LightGBM, enhancing our ability to interpret the models’ results accurately and efficiently. Thus, these algorithms not only allow us to model complex relationships within the data but also enable us to offer clear, detailed insights into how different factors influence these relationships.

The choice of these specific algorithms significantly impacts the study’s results. The gradient boosting mechanism inherent in XGBoost and LightGBM helps capture complex, non-linear relationships that might be overlooked by simpler algorithms. Their ability to handle missing data and accommodate categorical variables provides a comprehensive understanding of the dataset. The interpretability offered by the SHAP methodology provides a nuanced understanding of the variable importance, providing clear insights into each feature’s contribution to the predicted LST.

Despite its potential, only a handful of studies have adopted this methodology to investigate the driving factors of LST. For instance, Zhou et al. (2022) leveraged the XGBoost model and the SHAP method, among other techniques, to explore the relationship between urban landscape structure and LST [46]. Similarly, Kim et al. (2021) applied XGBoost and SHAP models to develop an LST prediction model for Seoul, South Korea [47]. Both studies underscored the significant influence of certain environmental factors on LST, thereby validating the utility of these ML algorithms in deciphering complex environmental relationships.

## 3. Results

This section will present the findings of the study, including the results of the spatiotemporal analysis of LST trends, 3D plots, GWR analysis, and ML analysis.

### 3.1. Visual Analysis of Spatiotemporal LST Trends

**Figure 3:**
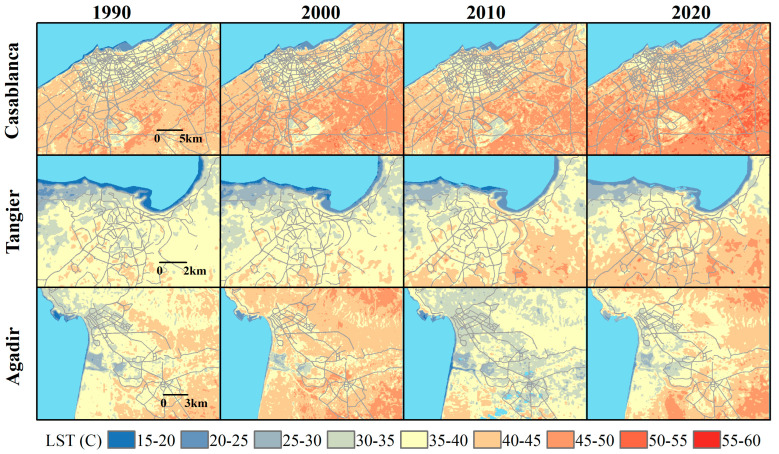
Temporal evolution of LST during summer (June–August) for coastal Moroccan cities (Casablanca, Tangier, and Agadir) from 1990 to 2020. The color gradient represents LST intervals from 15 to 60 °C, with blue indicating lower temperatures, yellow medium temperatures, and red higher temperatures.

**Figure 4:**
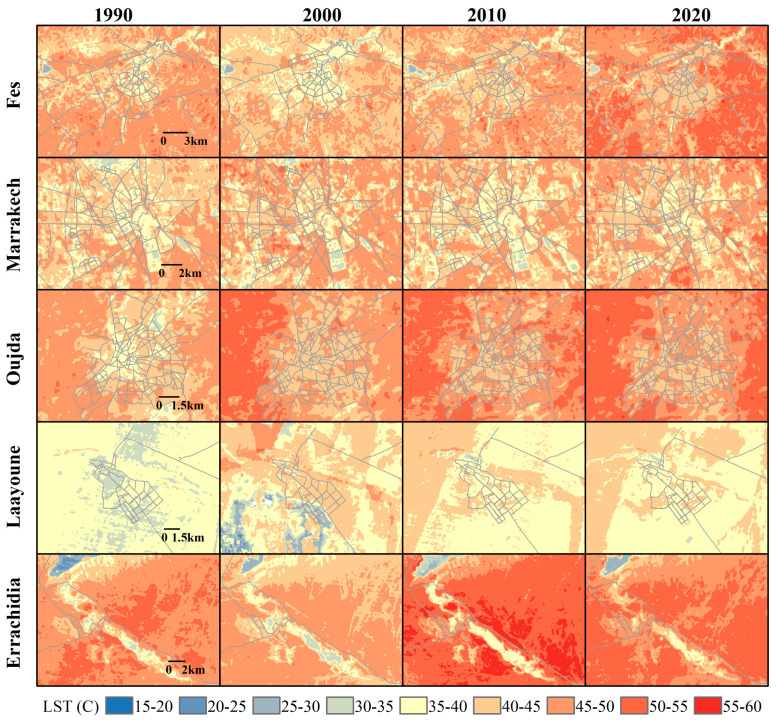
Temporal evolution of LST during summer (June–August) for inland Moroccan cities (Fes, Marrakech, Oujda, Laayoune, and Errachidia) from 1990 to 2020. The color gradient represents LST intervals from 15 to 60 °C, with blue indicating lower temperatures, yellow medium temperatures, and red higher temperatures.

The analysis of LST evolution in the target Moroccan cities, shown in Figure 3 (coastal cities) and Figure 4 (inland cities), reveals a general trend of increasing temperatures over the three-decade period (1990–2020), with some variations and exceptions. The satellite images used to extract the LST data were specifically gathered during the summer months of June, July, and August. This timing aligns with the post-harvest season for the most common crops in Morocco, wheat and barley [48]. During this period, fields are typically left fallow after harvest, which could potentially contribute to the bareness of the lands and consequently higher temperatures.

In coastal cities like Casablanca, Tangier, and Agadir, the urban areas generally experienced an increase in LST. Casablanca, the largest and most populous city with a large urban cover and intense industrial and commercial activities, showed a significant increase in LST in both urban and surrounding non-urban areas. The increasing trend of LST in the city could be attributed to the combination of rapid urbanization, a decrease in green spaces, and the replacement of natural landscapes with heat-absorbent concrete and asphalt. Additionally, the thermal properties of the built-up materials might also contribute to higher LST. The Bouskoura forest, the only significant green space in the vicinity of the city, is considered a vital element for the thermal regulation of the region. However, the observed increase in the LST values of the forest from 25–30 and 30–35 in the earlier years to 35–40 in 2020 might indicate a degradation of the forest cover or other changes in its ecological conditions. This could be attributed to factors such as deforestation, encroachment for urban development, or changes in moisture levels due to variations in precipitation or groundwater extraction.

Tangier, the northernmost coastal city with a Mediterranean climate and slightly hilly topography, also showed a similar trend to Casablanca, albeit at a slower rate, possibly due to a lower level of urbanization. However, the observed increase in LST in non-urban areas could be related to agricultural practices, land degradation, and potential deforestation. Notably, the decrease in LST over time in the Cap Spartel Natural Reserve and other parks suggests a reduction in their size or density, potentially driven by urban expansion or environmental degradation.

The trend in Agadir differs, with the city experiencing a more fluctuating LST pattern, which could be linked to its unique geographical setting. The city is close to the Anti-Atlas mountains, which could impact local weather patterns, and the city’s climate is influenced by both Atlantic marine effects and desert influences. The rise in LST in 2000 might have resulted from a prolonged dry period, leading to a decrease in vegetation cover and increasing surface exposure to solar radiation. The drop in 2010 could be related to a wet year, which may have caused increased vegetation density and soil moisture, both contributing to lower LST [3]. Furthermore, the golf courses are significant green spaces in the urban landscape and contribute to moderating the local thermal environment. Despite variations in LST in the city and surrounding areas, these golf courses consistently maintain lower LST values between 20–25 and 25–30, underlining their role as “cool islands”.

Inland cities like Fes, Marrakech, Oujda, Laayoune, and Errachidia also exhibited an increasing trend in LST, with some exceptions. In Fes, there was a decrease in LST values in 2000, which could be due to it being a wet year. Increased precipitation can lead to lower LST by increasing soil moisture and evapotranspiration.

Marrakech experiences a hot semi-arid climate, and its relatively stable LST through the years could be due to the city’s extensive green areas and irrigation practices. However, the gradual reduction in cool spots, especially in the north, might be related to changes in land use, urban expansion, and possibly changes in irrigation or agricultural practices and drought impacts.

For Laayoune, the inconsistent trend of LST might be influenced by its unique geographical and climatic characteristics, as it is situated on the border between desert and coastal climates. Periodic fluctuations in rainfall, possibly influenced by Atlantic and Saharan climatic systems, could cause significant changes in soil moisture and vegetation cover, leading to variable LST.

Finally, Errachidia showed the hottest LST values in 2010, with the coolest year being 2000, possibly a wet year. The observed variations in LST are likely tied to the city’s desert-like climate, where vegetation cover is minimal, and the land surface is extensively exposed to solar radiation. The differences in LST across years might reflect climate variability, with wetter years (e.g., 2000) leading to lower LST, and drier years (e.g., 2010) resulting in higher LST. The Al-Hassan Addakhil dam, acting as a local “cool island”, might affect the surrounding LST due to the cooling effect of water bodies.

### 3.2. The 3D Plots of LST Trends

In this subsection, we present the LST trends via 3D plots. The spectral indices—NDVI, NDBI, and NDWI—are scrutinized in relation to LST for two categories of Moroccan cities: coastal and inland, based on 3000 randomly generated points for each city.

**Figure 5:**
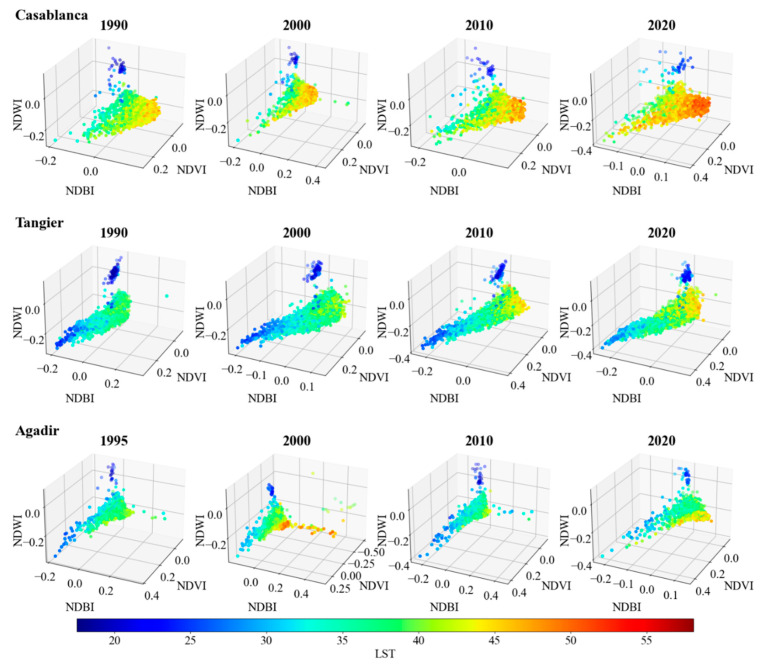
Spectral indices (NDVI, NDBI, NDWI) plotted against LST over time (1990–2020) for the coastal cities Casablanca, Tangier, and Agadir. The color of the dots represents LST using the jet colormap, with darker blue indicating lower temperatures and reddish colors indicating higher temperatures. Note: data for Agadir in 1990 is unavailable; hence, data from 1995 is used for initial representation.

Commencing with the coastal cities (Figure 5), the spectral indices of Casablanca, Tangier, and Agadir depict relatively consistent values across the observed years, while portraying a marked evolution in LST. More specifically, most points in Casablanca’s 1990 plot present LST values predominantly between 16 °C (represented by a dark blue color in the jet colormap) and 50 °C, with similar ranges of NDVI, NDBI, and NDWI. However, by 2020, the points exhibited a perceptible shift towards warmer temperatures, particularly where NDBI > 0 and NDVI < 0.1. Tangier and Agadir displayed comparable trends, with LST increasing notably in 2020 in most data points and the number of cooler points (blue-colored dots) with NDWI < 0 and NDBI < 0 decreasing.

**Figure 6:**
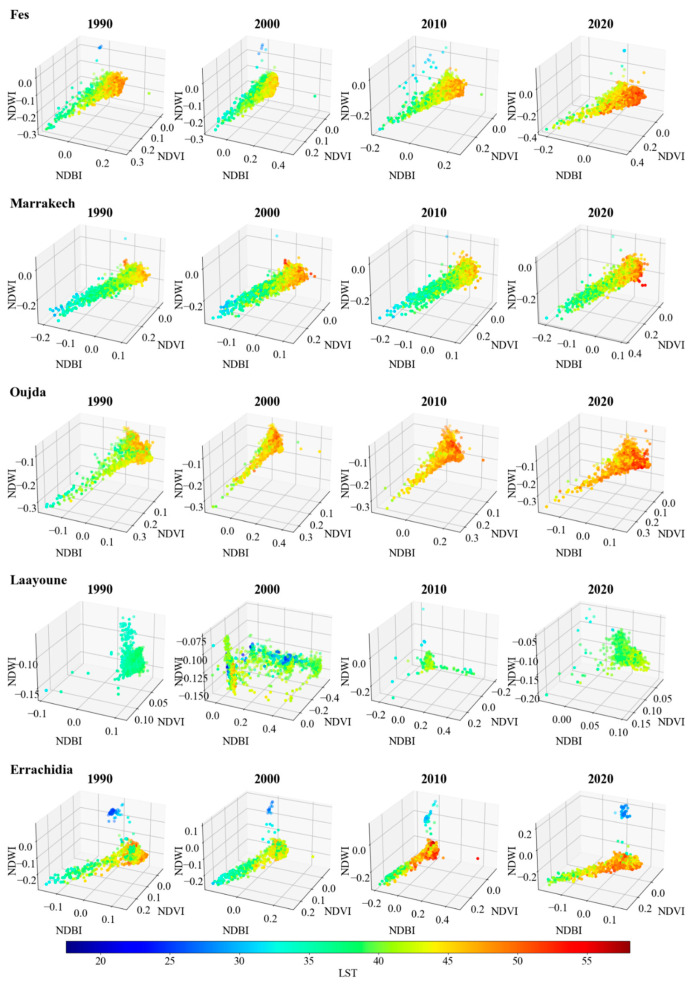
Spectral indices (NDVI, NDBI, NDWI) plotted against LST over time (1990–2020) for the inland cities Fes, Marrakech, Oujda, Laayoune, and Errachidia. The color of the dots represents LST using the jet colormap, with darker blue indicating lower temperatures and reddish colors indicating higher temperatures.

Switching focus to the inland cities (Figure 6), Fes, Marrakech, Oujda, Laayoune, and Errachidia, a similar trend in LST increase is observed over the years, although the evolution of spectral indices is more diverse. For instance, in Fes, despite a slight decrease in warmer points (orange and red-like colors) in 2000, by 2020, the number of such points had increased, especially those with higher NDBI values between 0.3 and 0.4.

Similarly, Marrakech showed a remarkable increase in warmer points in 2000 but a slight decrease in 2010. Nevertheless, by 2020, there was an alarming rise in the number of extremely warm points (LST of >55 °C), correlating with a decrease in the number of cooler points (LST of <20 °C). Oujda demonstrated a similar pattern, with an almost complete transformation to warmer points by 2020 and a significant reduction in cooler and medium-cooler dots. On the other hand, Laayoune exhibited a more erratic pattern across the years, particularly in 2000 and 2010, where the spectral indices and LST demonstrated quasi-random distributions. It is important to note that these fluctuations could have been influenced by the methodological approach where the mean annual images were computed using several cloud-masked images captured under different weather conditions, leading to a varied range of values.

The observed trends suggest a noticeable rise in LST over time, more pronounced in the inland cities. The trend aligns with previous studies indicating an increase in urban heat island effects, particularly in built-up and less vegetated areas, underscored by high NDBI and low NDVI values, respectively. Furthermore, the spectral indices and LST variations over time, particularly the NDBI increases and NDVI decreases, are congruent with the global increase in urbanized and built-up areas and the decrease in vegetated areas. NDWI portrays less-clear trends, implying a complex relationship between urbanization and LST. It is worth noting that the presence of water bodies, such as dams, can greatly influence the NDWI, as observed in Errachidia’s 1990 data points. In such instances, a lower temperature (LST < 25 °C) was observed, possibly due to the cooling effect of water bodies.

### 3.3. GWR Analysis

**Figure 7:**
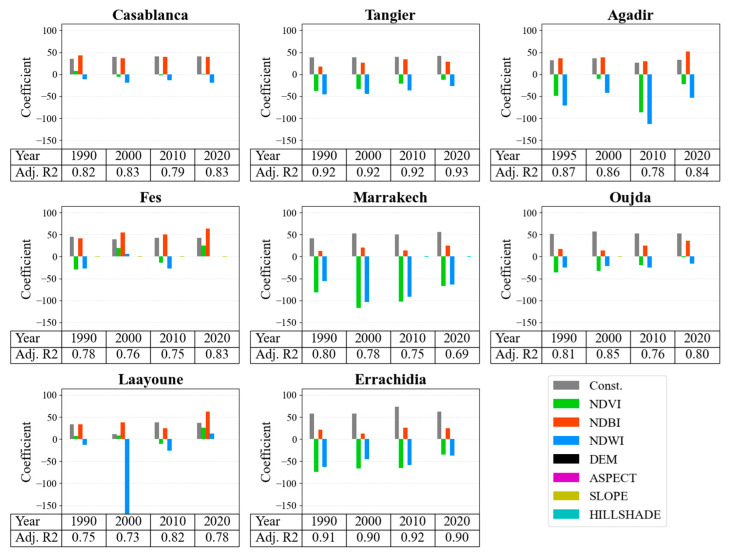
Bar plots illustrating temporal trends of GWR coefficients for spectral indices and geographical features across the target Moroccan cities for the years 1990, 2000, 2010, and 2020. The table below shows corresponding adjusted R-squared values, indicating model fit for each city and year. Note that DEM, ASPECT, SLOPE, and HILLSHADE are included in the legend for completeness, even though they may not be discernible in the figure due to their values.

The results of the GWR analysis provide quantitative insights into the relationships between LST and the selected factors. The results of the GWR analysis are shown in Figure 7, focusing on the spectral indices of three Moroccan coastal and inland cities across the years 1990, 2000, 2010, and 2020, which are presented in this section. The detailed results are listed in Appendix A, Table A2 and Table A3 for coastal and inland cities, respectively.

For Casablanca, the constant value shows a gradual increase over the years, indicating a potential rising trend in the LST. Among the three spectral indices, NDBI consistently demonstrated high mean values with a slightly decreasing trend in standard deviation over time. The NDVI values showed a shift from positive to negative during this period, with a decline in absolute values in the later years, indicating possible vegetation reduction. Interestingly, the NDWI values remained negative throughout, with relatively high standard deviation values, suggesting significant variations in water content. Other factors such as DEM, slope, aspect, and hillshade demonstrated minimal variation, with small standard deviation values indicating stability over time. The adjusted R^2^ values for Casablanca showed a slight increase over the years, indicating an improvement in model performance.

Similarly, in Tangier, the constant value revealed a marginal increase over the years, suggesting a potential rising trend in LST. The NDVI values were negative throughout, indicating a potential decrease in vegetation over time. The NDBI values showed a general increase, while the NDWI values were consistently negative but displayed a reduction in absolute values over time, implying potential changes in water bodies. Among the other factors, hillshade demonstrated a slight negative trend, while DEM, slope, and aspect showed minimal variations. The adjusted R^2^ values for Tangier were consistently high and exhibited a marginal increase over the years, showing an excellent and improving model performance.

In the case of Agadir, the constant value showed fluctuations but ended with a higher value in 2020 compared to 1990, indicating a potential overall increase in LST. The NDVI values were consistently negative and displayed significant fluctuations, suggesting major vegetation changes over time. Both the NDBI and NDWI values showed varying trends and high standard deviation values, indicating significant variations in built-up areas and water bodies. Among the other factors, only the hillshade showed a slight negative trend, while DEM, slope, and aspect demonstrated small variations. The adjusted R^2^ values for Agadir showed a decline in 2010 but an improvement in 2020, indicating some fluctuation in model performance over time.

In Fes, the constant values reveal a relatively fluctuating trend in the LST. NDVI values have shifted from negative to positive, suggesting significant changes in vegetation cover. A consistent rise in NDBI values might indicate an increase in built-up areas. Notably, NDWI values have also moved from negative to positive, signifying variations in water content. Among other factors, aspect and hillshade show a consistent negative trend, while DEM and slope values show minimal changes. The adjusted R^2^ shows an increase in 2020 after an initial decline, pointing to an improvement in the model’s predictive power.

Marrakech’s constant value shows a consistent rising trend, indicating an increase in LST over time. NDVI values are consistently negative and notably high, implying a major reduction in vegetation. The NDBI and NDWI values demonstrate fluctuating trends, implying significant changes in built-up areas and water bodies. The adjusted R^2^ value shows a decline, suggesting a decrease in the model’s performance over time.

In Oujda, the constant value shows a relatively stable trend. The NDVI values display an upward shift, suggesting an increase in vegetation. The NDBI values show a general increase, pointing to an expansion of built-up areas. Meanwhile, NDWI values, which are all negative, show a decrease in absolute value, suggesting an increase in water content. The adjusted R^2^ value shows fluctuations but ends with a relatively high value, indicating good model performance.

Laayoune’s constant value displays a rising trend after an initial decline in 2000. The NDVI values show major fluctuations, implying significant changes in vegetation. Similarly, NDBI and NDWI values demonstrate considerable fluctuations, suggesting substantial changes in built-up areas and water bodies. The adjusted R^2^ value shows an improvement in 2010 and remains relatively high, indicating that the model’s performance has improved over time.

In Errachidia, the constant value shows fluctuations but ends higher in 2020 compared to 1990, indicating a general increase in LST. The NDVI values are consistently negative, suggesting a decrease in vegetation over time. The NDBI and NDWI values demonstrate slight fluctuations, suggesting changes in built-up areas and water bodies. The adjusted R^2^ value remains relatively high, demonstrating good model performance.

### 3.4. ML Modeling and SHAP-Based Explanation

#### 3.4.1. ML Models’ Performance Assessment

Table 3 encapsulates the comparative performance of two machine learning models—XGBoost and LightGBM—across the eight Moroccan cities for the years 1990, 2000, 2010, and 2020. Each model’s predictive performance is evaluated using two standard metrics: the coefficient of determination (R^2^) and the root mean square error (RMSE). A higher R^2^ and a lower RMSE indicate superior predictive performance. As is evident, the XGBoost algorithm outperforms LightGBM in most cases, showcasing its robustness and efficacy in predicting urban expansion. Based on the comprehensive evaluation, XGBoost was subsequently chosen for further in-depth analysis.

#### 3.4.2. LST Driving Factors’ Importance

**Figure 8:**
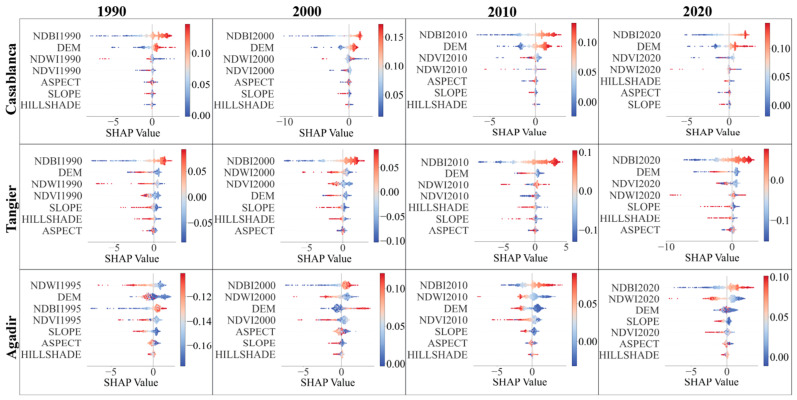
SHAP-value-based importance of LST driving factors in coastal Moroccan cities.

**Figure 9:**
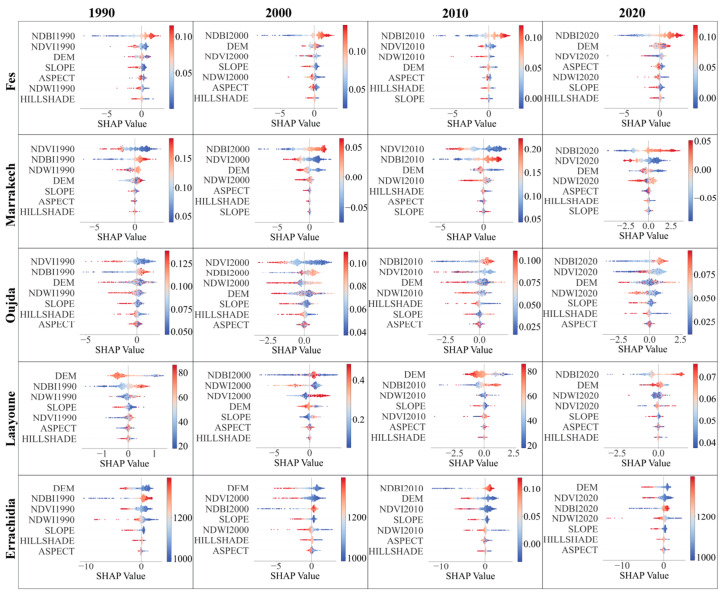
SHAP-value-based importance of LST driving factors in inland Moroccan cities.

We used SHAP summary plots to gain insights into the model’s inner workings for each city for every year. The results for coastal and inland cities are illustrated in Figure 8 and Figure 9, respectively.

Each plot illustrates the distribution of the effects each feature has on the model’s prediction. The position on the y-axis is determined by the feature and on the x-axis by the Shapley value. The color represents the value of the feature from low to high. Higher Shapley values, either in the positive or negative direction, indicate features that significantly influence the model’s prediction. It is worth noting that the magnitude and direction of these effects vary across different cities and years, reflecting the distinctive local environmental and temporal dynamics.

The primary feature influencing the prediction of LST across the target cities and years, according to the SHAP summary plots, is consistently the NDBI. However, this influence and the importance of other features vary across cities and over time.

For coastal cities, NDBI is shown to exert a significant influence in all cities and years, but the relationship and level of influence differ notably between the cities and across the decades. In Casablanca, a higher value of NDBI is associated with increased SHAP values, implying a positive correlation with LST. This pattern is consistent from 1990 to 2020, suggesting the ongoing urbanization contributing to the city’s increasing heat island effect. In contrast, Tangier and Agadir exhibit a more complex relationship between NDBI and LST. While NDBI remains a top contributing feature, the SHAP value ranges between positive and negative, suggesting localized differences in the built-up areas’ thermal properties.

The influence of the elevation (DEM) is more variable across the coastal cities. In Casablanca, DEM consistently ranks as the second most important feature, with a positive correlation with LST. This effect is less pronounced in Tangier, where DEM’s importance fluctuates over time. Meanwhile, in Agadir, the prominence of DEM increases over time, replacing NDWI as the second most important feature by 2020.

Moving on to the inland cities, NDBI’s importance as a feature remains consistent but exhibits different patterns from coastal cities. Fes shows a positive correlation between NDBI and LST similar to Casablanca. Marrakech and Oujda, however, reveal a fluctuating importance and relationship between NDBI and LST, similar to what was observed in Tangier and Agadir.

Comparing the importance of features between the coastal and inland cities, it appears that the NDBI and DEM generally have more influence in coastal cities, possibly due to the higher degree of urbanization and variations in terrain height affecting the heat distribution. In contrast, in inland cities, NDVI also emerges as a critical feature, potentially due to vegetation’s role in regulating temperature in these areas.

The changes over time also suggest the ongoing urbanization and climate change’s potential impact. The increasing importance of NDBI in most cities points to the increasing role of built-up areas in shaping urban heat. The shifting influence of NDVI and NDWI also implies potential changes in vegetation and water bodies’ roles in modulating the urban temperature.

In summary, this comparative study reveals significant heterogeneity in LST’s influencing factors across different cities and over time, reflecting the complexity of urban thermal dynamics. These findings underscore the importance of considering local context and temporal changes when modeling and managing urban heat.

#### 3.4.3. Factors’ Interactions and LST Prediction

**Figure 10:**
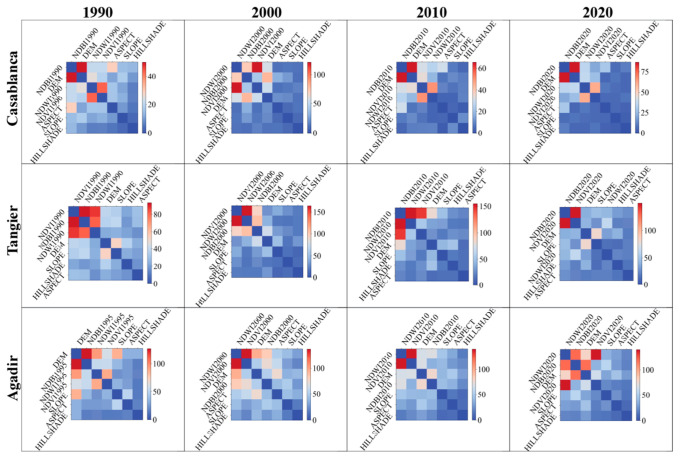
Heatmap plots illustrating the interactions of different features influencing LST across coastal Moroccan cities from 1990 to 2020. The intensity of color represents the interaction score, with higher values indicating stronger interactions. Notable interactions are discussed in the text.

**Figure 11:**
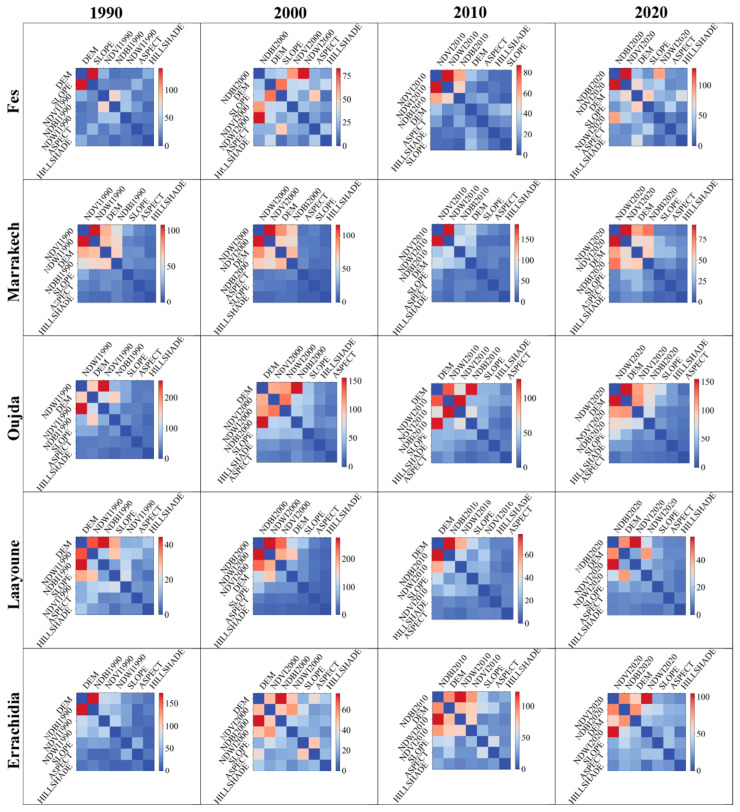
Heatmap plots illustrating the interactions of different features influencing LST across inland Moroccan cities from 1990 to 2020. The intensity of color represents the interaction score, with higher values indicating stronger interactions. Notable interactions are discussed in the text.

In addition to the factors’ importance, we examined the interactions between the most significant factors that affect LST prediction. In Figure 10 and Figure 11, we show the interactions of the factors in a series of heatmap plots for the target cities, ranging from the years 1990 to 2020. Herein, the discussion focuses on the notable interactions, which are defined as colored in reddish colors.

In Casablanca, across the years, the interactions between DEM and NDBI have been consistently prominent, highlighting the significant interplay between the city’s topography and urbanization in affecting the LST. Notably, in 2000, a strong interaction between NDVI and NDWI also emerged, suggesting the potential synergistic role of vegetation and water bodies in modulating the city’s temperature.

The factors’ interactions in Tangier exhibit more variation over time. In all years, interactions involving NDVI, NDWI, and NDBI are predominant. This implies a complex interplay between vegetation, water, and built-up areas in shaping the city’s heat dynamics. In addition, the interactions between SLOPE and DEM in 1990 and 2020 emphasize the city’s topography role in its thermal patterns.

The heatmaps for Agadir highlight the dominant interaction between NDBI and DEM, especially in 1990 and 2020, with NDWI also showing strong interactions with DEM, NDVI, and NDBI in different years. This reflects the interweaving effects of urbanization, elevation, vegetation, and water bodies on Agadir’s urban heat.

In Fes, the key interactions change over time. The interaction between SLOPE and DEM in 1990 reveals the topography’s importance. Yet, in the subsequent years, interactions involving NDVI, NDWI, and NDBI become more prevalent, echoing the complex role of urbanization, vegetation, and water bodies in inland cities’ heat dynamics.

Marrakech shows strong and consistent interactions between NDVI and NDWI across all years, underscoring the combined role of vegetation and water bodies. Interactions involving DEM also stand out, suggesting the city’s critical role in topography.

The heatmaps for Oujda exhibit several strong interactions, particularly between NDWI and NDVI in 1990, and DEM’s interactions with other features in subsequent years. These patterns reflect the city’s complex heat dynamics, intertwining vegetation, water bodies, urbanization, and topography.

The heatmap for Laayoune highlights the significant interaction between NDBI and NDWI in 2000. This suggests a critical interplay between urbanization and water bodies. Moreover, the interactions of DEM with NDBI, NDWI, and NDVI in different years underline the significant role of the city’s terrain.

In Errachidia, NDBI’s interactions, particularly with DEM, are predominant in all years. This emphasizes the intertwining effects of urbanization and the city’s topography. Notably, in the year 2000 and onwards, interactions involving NDVI, NDWI, and DEM also become prevalent, suggesting the role of vegetation and water bodies, alongside urbanization and topography, in shaping the city’s thermal dynamics.

In summary, these heatmaps uncover the complexity of feature interactions influencing LST across different Moroccan cities. The interactions vary across cities and over time, echoing the cities’ evolving urban form, land use, and climate conditions.

#### 3.4.4. Analysis of the Association between Potential Factors in LST Prediction

**Figure 12:**
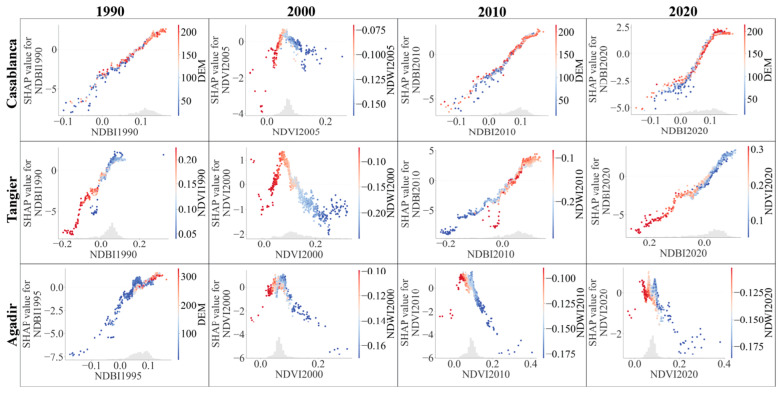
Dependence plots illustrating the most notable interactions between different factors influencing LST across coastal Moroccan cities from 1990 to 2020.

**Figure 13:**
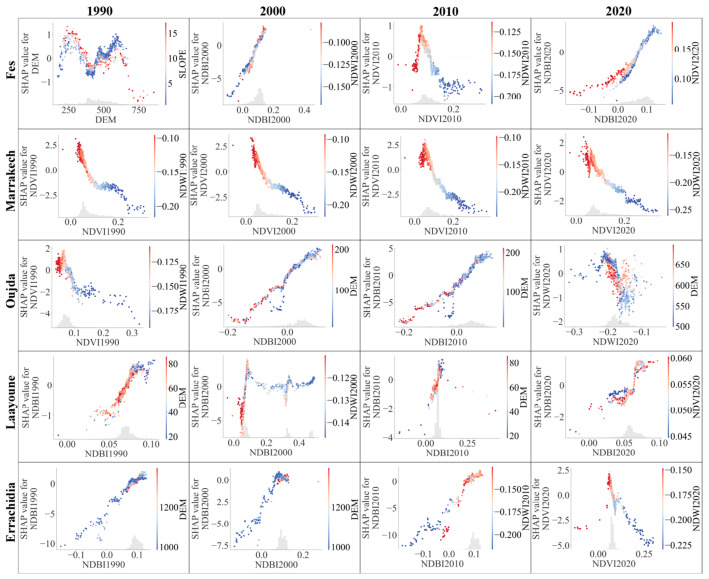
Dependence plots illustrating the most notable interactions between different factors influencing LST across inland Moroccan cities from 1990 to 2020.

The present section delves deeper into the relationships between the most prominent features influencing LST across the selected Moroccan cities. Specifically, we present a series of scatter plots that visualize the most notable interactions identified from the preceding heatmaps for each city from 1990 to 2020. These scatter plots illustrated in Figure 12 and Figure 13 provide a more detailed perspective on the pairwise interactions between variables, highlighting the trends and correlations in a more focused manner. By studying these plots, we can gain more profound insights into how these interactions have evolved over time and their role in influencing LST in different geographical contexts—coastal versus inland cities.

In the case of Casablanca, we can observe a clear temporal evolution in the interactions between NDBI and its SHAP values. In 1990, a linear relationship was present with histogram bars concentrated around an NDBI value of 0.1. Over the next three decades, this relationship evolved to quasi-linear, while the concentration of histogram bars gradually shifted towards higher NDBI values. There was a noticeable difference in the distribution of reddish dots (DEM of 100–210) and blue dots (0–125), which might indicate changes in the urban structures and infrastructures of the city.

The interaction between NDBI and SHAP values in Tangier showed a shift from the situation in 1990, where histogram bars of NDBI were concentrated between 0 and 0.1, to 2020, where the concentration was between −0.1 and 0.1. This trend, along with the change in the distribution of red and blue dots, signifies potential environmental shifts, possibly due to the city’s development or environmental policies over the three decades.

Agadir exhibited an intriguing progression, with a linear relationship between NDBI and its SHAP values in 1990 evolving to a more concentrated distribution of NDVI by 2020. The increasing density of blue dots over time might signify changes in water distribution, possibly due to urbanization and climate change.

Fes presented a unique quasi-sinusoidal relationship between DEM and SLOPE in 1990, which shifted to a quasi-linear relationship by 2020. The evolving interaction of these variables signifies significant changes in the city’s topography and infrastructural development.

Marrakech exhibited a quasi-linear relationship between NDVI and SHAP values across the decades. However, the distribution of red and blue dots changed, indicating potential shifts in vegetation and water indices. This could be an outcome of land use changes or responses to climate variations.

Oujda’s scatter plots show evolving interactions between NDBI and SHAP values over the years. The significant shift in the concentration of histogram bars and the distribution of red and blue dots across DEM values might indicate changes in the city’s urban development and topographical features.

Laayoune demonstrated an intricate pattern over the years. From a concentration of NDBI histogram bars between 0.05 and 0.1 in 1990, the city showed a more varied pattern by 2020. The fluctuation in SHAP values might result from changes in land use and environmental management over time.

Errachidia’s scatter plots reveal a shift in the interactions between NDBI and SHAP values over the years. The concentration of histogram bars and the distribution of red and blue dots across DEM values suggest significant changes in the city’s urban development and topographical features.

## 4. Discussion

### 4.1. LST Trends and Contributing Factors

General observations in all assessed cities indicate that urban areas often manifested as UHSs with cooler LST, especially in coastal cities (i.e., Casablanca, Tangier, and Agadir). This is similar to global trends observed in various Mediterranean, arid, and desert cities like Erbil [49], Cairo [4], Dubai [50], Abu Dhabi [50,51], and Tehran [52]. Over time, however, these UHSs have been declining. Indeed, the results consistently revealed an increase in LST across all cities regardless of their type, mainly due to urbanization and degradation of vegetation cover. This trend is more pronounced in inland cities surrounded by barren landscapes, notably Errachidia, Oujda, Fes, and Marrakech.

#### 4.1.1. Coastal Cities

Coastal effects significantly influence LST. For instance, Al-Ruzouq et al. (2022) found that daytime LST values in coastline districts were lower than those further inland in a study conducted in the arid coastal cities of the United Arab Emirates [53]. This observation aligns with the findings of Peng et al. (2021), who reported a significant cooling effect of the sea during the daytime in the Japanese prefecture of Fukuoka [54]. Despite these coastal cooling effects, a rise in LST values was observed in Casablanca, Tangier, and Agadir between 1990 and 2020. GWR and ML analyses identified NDBI as a potent influencer of LST across all coastal cities. The built-up index’s high SHAP values, suggesting a positive correlation with LST, point towards the potent role of ongoing urbanization in driving the rise in LST. The consistent interactions between NDBI and DEM across the decades underline the significant interplay of topography and urbanization in affecting LST. This interaction is particularly pronounced in 2020, hinting at the possible synergistic role of vegetation and water bodies in mitigating city temperature.

This LST increase was particularly noticeable in Casablanca—the economic capital and the most populous city of the country, where an extensive urban sprawl has taken place. The city witnessed a 38.32% increase in the built-up area between 2000 and 2015 [7], leading to the loss of green spaces and the emergence of scattered UHIs [10,55]. These UHIs are observed mainly in new industrial zones scattered across the city. This expansion occurred at the expense of green spaces. The Bouskoura forest, an example of green urban space on the outskirts of the city, displayed an increase in LST values over time from LST values ranging between 20 and 40 °C (1990) to values ranging between 35 and 50 °C (2020), which may indicate potential degradation or changes in its ecological conditions as a result of a mix between weather extremes [56] and anthropogenic interventions [10,18,19,55].

In Tangier—the country’s second industrial hub with several industrial parks surrounding the city [57]—the LST trends bear resemblance to those observed in Casablanca, albeit at a slower pace potentially due to a lesser degree of urbanization and the existence of blue-green spaces (i.e., Atlantic Ocean, Cap Spartel Nature Reserve, golf courses, and parks) to the north and northwest of the city that provide a cooling effect [12]. However, the observed increase in LST over time in the Cap Spartel Nature Reserve and other parks hints at a potential reduction in their size or density, which may be corroborated by the World Cities Report’s data showing a 43.71% increase in the city’s built-up area from 2000 to 2015 [7].

Agadir presented a more fluctuating LST pattern, potentially due to its geographical setting being at the foot of the Atlas mountains with changing green cover [58], Souss River at the south providing cooling effects, and variable climatic conditions, including periods of drought and high rainfall that affect vegetation and soil moisture. Historically, the years 2000 and 2001 were the years with the lowest rainfall amounts in Morocco for the period 1984–2016 [56]. This could explain the high LST values recorded for Agadir in 2000. Surprisingly, despite 2010 being the warmest year in the period 1984–2018, yet with a higher total precipitation anomaly [56], cool LST values were observed across the city and its surrounding area. The year 2010 was a year of high precipitation and lower LST values, and it is possible that the higher rainfall led to increased vegetation cover, which can lower LST through evapotranspiration. Additionally, the increased cloud cover associated with higher rainfall can reduce the amount of sunlight reaching the surface, thus reducing surface temperatures.

#### 4.1.2. Inland Cities

In inland cities, the increase in LST especially is more pronounced in urban and non-urban areas alike. The city of Fes exemplified an escalating trend in LST, registering some of the apex values in the year 2020. This trend was punctuated by a transient decline in the year 2000 observed across the region, potentially attributed to an escalation in precipitation. Urban areas manifested diminished LST values, reaping the cooling benefits of green spaces interspersed within the cityscape, the Fes River that courses through the city, and the utilization of specific construction materials such as mud and adobe in the ancient medina. These materials are renowned for their thermal insulation properties [22,59]. Nonetheless, the proliferation of urban zones and the expanse of green spaces, as indicated by GWR and ML analyses, corroborated by data asserting that the city underwent a 24.32% expansion from 2000 to 2015 [7], signal substantial urban advancement. Consequently, the city appears to be on a trajectory toward a warming phase [56].

Marrakech maintained relatively stable LST over the years, potentially as a result of extensive green areas and irrigation practices across the city and its surroundings including the historic Agdal and Menara gardens to the south in addition to golf courses and resorts with green spaces to the east. This aligns with the results presented in [9], which found the mix and amount of vegetation to be an important modulator of surface temperature and it can be used as a natural mitigation mechanism to reduce excess urban heating in Marrakech. It is also observable that LST values are lower inside the city than in its peripheral areas, creating UHSs in accordance with [12]. Despite the LST stability over the years, however, a gradual reduction in cool spots indicated possible changes in land use, such as urban expansion as noted in [9], modification in agricultural practices, and impacts from periodic droughts.

Oujda, like Fes and Errachidia, had some of the highest LST values recorded in 2020. The urban and surrounding areas showed an increasing trend in LST over time, potentially due to urban expansion and transformation of land use. In fact, the author in [56] found that Oujda is one of two cities that have a 5% statistically significant level of decreasing total precipitation anomaly and a standard precipitation index with values inferior to −12 and −0.3, respectively. The city was built in a circumscribed space surrounded by desert barren lands. Similar to Marrakech, UHSs are observed across urban areas, a phenomenon more pronounced in 1990 and less pronounced in 2020.

Errachidia, characterized by a desert-like climate, exhibited the hottest LST values among all cities in 2010. The coolest values were observed in 2000. Similar to Fes, Marrakech, and Oujda, the city also exhibited UHSs with low LST observed in urban areas benefiting possibly from the cooling effects of the M’daghra forest and the nearby Al-Hassan Addakhil dam [24].

In contrast to other cities, Laayoune exhibited an inconsistent LST trend especially in the year 2000, influenced by a unique combination of factors. GWR and ML analyses have indicated that the NDBI and DEM play a significant role in influencing LST. The city’s unique location close to the Atlantic Ocean, the intermittent lake to the north, and the sandy surroundings contribute to this complexity. The climatic and soil characteristics further compound these factors, where shifts in rainfall create considerable variations in soil moisture and vegetation cover, and subsequently in LST. These intricate dynamics, involving natural and human-induced factors, contribute to an LST trend that may not immediately seem intuitive.

### 4.2. Methodological Implications

The utilization of ML modeling, specifically the application of XGBoost and LightGBM, and SHAP-based explanation in this study has proven effective in analyzing the LST trends across various Moroccan cities. These methods facilitated a robust and nuanced understanding of the spatiotemporal dynamics of urban heat, highlighting the role of spectral indices and terrain characteristics. Interestingly, in many cases, XGBoost outperformed LightGBM. Our methodology parallels other studies that have successfully employed similar techniques in analyzing urban heat dynamics [47]. Furthermore, the use of the SHAP-based explanation for interpreting the ML models has lent additional transparency and interpretability to the study, an aspect that aligns with recent calls for explainable AI [60].

Nonetheless, the use of ML models inherently comes with some challenges, such as the need for large and high-quality datasets and the complexity of model tuning. These challenges emphasize the importance of integrating ML with traditional statistical methods, a blended approach that could form an interesting direction for future studies.

The study’s reliance on satellite data for LST analysis is another significant methodological aspect. Satellite data provides a large-scale and longitudinal perspective that is invaluable for studying long-term trends in urban heat. However, there are notable limitations, including the coarse spatial resolution and potential accuracy issues [61]. It is also critical to account for the influence of atmospheric effects and sensor calibration on the LST values derived from satellite data [62].

The methodological approach used in this study contributes to the field of urban heat research by offering a scalable and comprehensive method for analyzing urban heat dynamics over space and time. However, future studies should consider complementing satellite data with ground-based measurements to improve data resolution and accuracy. Additionally, the integration of different modeling techniques can be further explored to provide more nuanced insights into urban heat dynamics.

### 4.3. Policy Implications

The findings of this study have significant implications for urban planning, particularly regarding the mitigation of the UHI effect. Our results highlight the importance of green-blue spaces in moderating LST, resonating with previous studies that underscore the cooling effects of urban vegetation and water bodies [3]. Consequently, urban planning policies should prioritize the preservation of existing green spaces and the creation of new ones to attenuate the UHI effect. Moreover, the adoption of sustainable building materials and designs that minimize heat absorption could further contribute to the reduction in urban heat [22,59]. Promoting sustainable urban practices such as green roofing, using reflective or permeable pavements, and urban forestry should also be considered vital components of urban planning strategies.

The escalating LST trend also entails critical public health implications. Increased urban temperatures have been associated with a surge in heat-related illnesses, particularly during the summer months [63] among elderly people [64]. In this light, public health strategies should be integrated with urban planning efforts to mitigate the health risks associated with urban heat. Furthermore, elevated temperatures in urban areas increase the demand for cooling, leading to higher energy consumption and, consequently, heightened carbon emissions [65]. Hence, energy-efficient solutions should be part of policy frameworks to alleviate the energy demand and curb carbon emissions.

Lastly, the study’s findings significantly contribute to climate change mitigation and adaptation strategies, particularly amid the intensifying indications of our planet, and, specifically, in regions like Morocco, trending towards a drying climate [14,56,66]. The preservation and enhancement of vegetation and water bodies in urban landscapes, as evidenced by the study, play a crucial role in moderating LST. Thus, strategies that promote the integration of these natural elements in urban landscapes should be given priority in climate adaptation planning. Urban planning should aim to balance built environments with natural ecosystems, leading to cities that are not only resilient to climate change impacts but also sustainable for future generations.

### 4.4. Limitations and Future Works

Despite its insightful findings, this study acknowledges certain limitations. Predominantly, the analysis considered data only from four discrete years across three decades (1990, 2000, 2010, and 2020), potentially neglecting important inter-annual, seasonal, and diurnal variations in LST dynamics. Future research should therefore aim to incorporate more comprehensive data, possibly using time series satellite images across varied seasons and day–night cycles (e.g., using MODIS data), to better understand the complexity of LST changes and inform urban planning and climate adaptation strategies.

Secondly, the findings of this study are influenced by specific weather events and climatic conditions of the selected years, such as exceptionally dry or wet years. These conditions could considerably impact the LST values, and their influence should be controlled in future analyses. Therefore, future studies should consider conducting analyses that incorporate the effects of weather events and climate anomalies.

The study also relied on satellite data for LST analysis. While satellite data offers a large-scale, synoptic perspective, it has inherent limitations concerning spatial resolution and potential accuracy issues. It also lacks the site-specific details that ground-based measurements could provide [67]. Therefore, future research could consider integrating ground-based measurements with satellite data to ensure higher data resolution and accuracy.

As for the avenues of future research, the study’s findings suggest intriguing possibilities. The relationships between spectral indices, terrain characteristics, and LST, as highlighted in our findings, warrant further exploration. There is a need to understand how these variables interact under different geographical and climatic contexts, not only in Moroccan cities but in other regions as well. These investigations could involve more diverse and specific terrain characteristics as well as landscape metrics, potentially revealing more nuanced aspects of their influence on LST.

Additionally, given the key role of urbanization in increasing LST, future research could also focus on how different urban development patterns and strategies influence urban heat. This could provide valuable insights for urban planning and climate adaptation strategies.

## 5. Concluding Remarks

This investigation conducted an in-depth temporal examination of summertime LST trends across Moroccan coastal and inland cities over three decades (1990–2020). Results revealed consistent increases in LST over time, with higher increases evident in inland cities compared to coastal ones. UHS features, predominantly seen in coastal cities, have been waning due to factors such as urbanization and the degradation of vegetation cover. This study has underscored the significance of urban design and land use in affecting LST, with built-up areas, particularly industrial zones, contributing significantly to rising temperatures. Notably, areas with green-blue spaces demonstrated mitigating effects on LST, emphasizing their importance in urban planning strategies.

The use of ML models, particularly XGBoost and LightGBM, in conjunction with SHAP-based explanations, has proven valuable for the nuanced interpretation of the data. However, ML models’ reliance on large, high-quality datasets and complex model tuning highlights the need for integrating traditional statistical methods such as GWR used in this study.

The implications of the rising LST trend are multifaceted. From an urban planning perspective, it underlines the critical need to prioritize green-blue spaces, utilize sustainable building materials, and adopt urban practices that mitigate the UHI effect. From a public health viewpoint, the rise in LST can lead to increased heat-related illnesses and demands for cooling, necessitating the incorporation of energy-efficient solutions and the integration of health strategies into urban planning. Lastly, in the broader context of climate change mitigation and adaptation, the findings emphasize the importance of integrating natural ecosystems into urban landscapes.

Despite its contributions, this study acknowledges certain limitations, primarily the use of data from only four discrete years, thereby overlooking inter-annual, seasonal, and diurnal variations in LST dynamics. It also acknowledges the impact of specific weather events and climatic conditions on the findings. Future studies should consider incorporating more comprehensive time series data and controlling for weather and climate anomalies. The integration of ground-based measurements with satellite data and the exploration of various modeling techniques would also enhance the understanding of urban heat dynamics.

## Figures and Tables

**Table 1:** A descriptive list of studies that dealt with the thermal environment in Morocco.

Study	Study Area(s)	Study Period	Goal	Highlights
[8]	Casablanca	2011	Estimate the ground temperature and evaluate the impact of vegetation on cooling the ground temperature	Found a strong correlation between vegetation cover and cold areas
[9]	Marrakech	2010/2011	Assess the impact of urbanization on surface climate	Found that the growing season surface temperature differences between urban and other cover types varied significantly
[10]	Casablanca	1984–2016	Highlight and monitor the spatial distribution of SUHI	Found that the seasonal cycle of daytime SUHI in the Casablanca region is different from other mid-latitude cities
[11]	Several Moroccan urban areas	2013	Carry out a large-scale assessment of UHI and reflect on its mitigation	Found a pronounced UHI in urban areas built within green lands and a UHS in cities built within arid zones
[12]	Five Moroccan cities	2016	Assess the impact of land cover on the UHI and UHS	Found multiple causes defining the different forms and amplitudes of the UHI
[13]	Marrakesh	1985–2020	Investigate, in the daytime, the relationship between green surfaces, built-up areas, and the SUHI	Found a maximum mean LST difference of 3.98 °C across the different city neighborhoods

**Table 2:** Descriptive list of the characteristics of target Moroccan cities.

	City	Population 2014 ^1^	Climate Zone ^2^	Elevation (m)	Built-up Change (%) 2000–2015 ^3^
Coastal	Casablanca	3,359,818	Hot-summer Mediterranean (Csa)	0	38.32
	Tangier	1,065,601	Hot-summer Mediterranean (Csa)	21	43.71
	Agadir	600,599	Hot semi-arid (BSh)	74	NA
Inland	Fes	1,150,131	Hot-summer Mediterranean (Csa)	409	24.32
	Marrakech	1,330,468	Hot semi-arid (BSh)	466	62.33
	Oujda	506,274	Hot semi-arid (BSk)/Cold desert (BWk)	540	36
	Laayoune	238,096	Hot desert (BWh)	64	NA
	Errachidia	418,451	Hot desert (BWh)/Cold desert (BWk)	972	NA

^1^ The population data for all cities, excluding Laayoune and Errachidia which are provincial, are based on the prefectural data of the 2014 population Census [16]. ^2^ Climate zones are determined based on (Beck et al., 2018)’s classification for the present-day (1980–2016) conditions [14]. ^3^ Built-up changes (2000–2015) extracted from the World Cities Report 2020 [7].

**Table 3:** Comparative Performance Metrics of XGBoost and LightGBM Algorithms Across the Target Moroccan Cities.

Algorithm	City	R^2^				RMSE			
		1990	2000	2010	2020	1990	2000	2010	2020
XGBoost	Casablanca	0.785	0.802	0.742	0.789	1.580	1.621	1.878	1.737
	Tangier	0.842	0.834	0.823	0.818	1.564	1.720	2.108	2.019
	Agadir	0.707	0.729	0.655	0.701	1.969	1.935	2.055	2.254
	Fes	0.714	0.660	0.673	0.783	1.709	1.731	1.635	1.588
	Marrakech	0.728	0.672	0.710	0.599	1.769	2.530	2.095	2.483
	Oujda	0.622	0.557	0.643	0.648	1.814	2.317	1.600	1.758
	Laayoune	0.579	0.576	0.673	0.554	0.816	2.996	1.005	1.011
	Errachidia	0.857	0.863	0.883	0.843	1.709	1.161	1.688	1.436
LightGBM	Casablanca	0.778	0.792	0.726	0.786	1.606	1.662	1.934	1.750
	Tangier	0.841	0.829	0.821	0.827	1.569	1.749	2.120	1.968
	Agadir	0.706	0.730	0.634	0.693	1.974	1.934	2.116	2.284
	Fes	0.715	0.643	0.640	0.762	1.707	1.775	1.717	1.665
	Marrakech	0.725	0.683	0.708	0.594	1.781	2.490	2.100	2.498
	Oujda	0.636	0.576	0.658	0.627	1.782	2.265	1.566	1.810
	Laayoune	0.590	0.567	0.673	0.544	0.805	3.028	1.006	1.022
	Errachidia	0.858	0.861	0.883	0.841	1.700	1.170	1.688	1.442

## Data Availability

Not applicable.

## Abbreviations

ANN	Artificial Neural Network
B10	Band 10 (in remote sensing, it refers to a specific spectral band of the satellite sensor)
B6	Band 6 (similar to above, another specific spectral band)
DEM	Digital Elevation Model
ETM	Enhanced Thematic Mapper (a sensor onboard Landsat satellites)
GBM	Gradient Boosting Machine (a machine learning algorithm)
GEE	Google Earth Engine
GIS	Geographic Information System
GWR	Geographically Weighted Regression
LST	Land Surface Temperature
LULC	Land Use Land Cover
ML	Machine Learning
MODIS	Moderate Resolution Imaging Spectroradiometer
NDBI	Normalized Difference Built-up Index
NDVI	Normalized Difference Vegetation Index
NDWI	Normalized Difference Water Index
NIR	Near Infrared (a type of light invisible to the human eye, used in remote sensing)
OLI	Operational Land Imager (a sensor onboard Landsat satellites)
R^2^	Coefficient of determination
RF	Random Forest
RMSE	Root Mean Square Error
SHAP	SHapley Additive exPlanations
SRTM	Shuttle Radar Topography Mission
SUHI	Surface Urban Heat Island
SVM	Support Vector Machine
SWIR	Shortwave Infrared (a specific region of the infrared spectrum used in remote sensing)
TIRS	Thermal Infrared Sensor (a sensor onboard Landsat satellites)
TM	Thematic Mapper (a sensor onboard Landsat satellites)
UHI	Urban Heat Island
UHS	Urban Heat Sink
UNESCO	United Nations Educational, Scientific and Cultural Organization

## Appendix A

**Table A1:** Hyperparameters explored in the grid search for the XGBoost and LightGBM models.

Algorithm	Hyperparameters	Description	Values Explored
XGBoost	learning_rate	Controls the learning process’s speed	0.01, 0.1
	max_depth	Maximum depth of the trees	3, 5, 7, 10
	min_child_weight	Minimum sum of instance weight needed in a child	1, 3, 5
	subsample	Subsample ratio of the training instances	0.5, 0.7
	colsample_bytree	Subsample ratio of columns when constructing each tree	0.5, 0.7
	n_estimators	Number of boosted trees to fit	100, 200, 500
	objective	Specifies the learning task and the corresponding learning objective	reg:squarederror
LightGBM	learning_rate	Controls the learning process’s speed	0.01, 0.1
	num_leaves	Maximum tree leaves for base learners	31, 127
	reg_alpha	L1 regularization term on weight	0.1, 0.5
	min_data_in_leaf	Minimum amount of data needed in a leaf	30, 50, 100, 300, 400
	lambda_l1	L1 regularization term on weights	0, 1, 1.5
	lambda_l2	L2 regularization term on weights	0, 1

**Table A2:** GWR results in the coastal cities.

City	Factor	1990		2000		2010		2020	
		Mean	Std.	Mean	Std.	Mean	Std.	Mean	Std.
Casablanca	Const.	35.692	5.018	39.605	4.386	40.404	4.948	40.944	4.811
	NDVI	7.033	23.826	−6.349	26.545	−2.997	29.032	0.461	27.232
	NDBI	42.543	11.784	36.56	10.979	39.572	11.006	39.998	7.857
	NDWI	−10.951	30.554	−18.647	34.765	−13.854	31.778	−19.335	32.538
	DEM	0.011	0.019	0.01	0.013	0.012	0.013	0.014	0.018
	Slope	0.000	0.001	0	0.001	0	0.001	0.001	0.001
	Aspect	0.026	0.078	−0.023	0.129	0.017	0.092	0.015	0.08
	Hillshade	−0.009	0.016	−0.017	0.018	−0.019	0.017	−0.018	0.018
	Adjusted R^2^	0.822		0.826		0.794		0.831	
Tangier	Const.	39.026	5.863	38.813	4.948	39.808	6.513	42.169	5.387
	NDVI	−38.218	30.132	−34.014	36.432	−21.487	65.482	−12.723	51.227
	NDBI	17.798	13.028	26.053	12.169	34.511	14.26	28.632	11.91
	NDWI	−45.8	42.133	−44.822	47.328	−36.657	70.21	−26.445	58.29
	DEM	−0.004	0.007	−0.007	0.008	−0.009	0.01	−0.01	0.009
	Slope	0	0.002	0	0.002	0	0.002	0.001	0.002
	Aspect	−0.025	0.055	−0.029	0.051	−0.025	0.062	−0.034	0.048
	Hillshade	−0.025	0.011	−0.024	0.011	−0.021	0.016	−0.022	0.013
	Adjusted R^2^	0.924		0.925		0.922		0.925	
Agadir *	Const.	31.38	4.597	35.987	5.182	26.795	4.528	32.715	5.51
	NDVI	−48.684	43.373	−9.77	60.739	−86.277	38.917	−21.971	66.192
	NDBI	36.725	17.395	38.461	25.28	29.707	15.674	52.079	20.057
	NDWI	−70.985	47.422	−42.9	57.859	−113.698	45.057	−53.55	65.415
	DEM	0.005	0.029	0.012	0.037	−0.002	0.027	0.004	0.026
	Slope	−0.002	0.003	−0.002	0.003	−0.002	0.002	−0.002	0.002
	Aspect	−0.022	0.067	−0.022	0.096	−0.005	0.106	−0.029	0.092
	Hillshade	−0.006	0.016	−0.009	0.018	0.002	0.019	−0.003	0.02
	Adjusted R^2^	0.873		0.855		0.784		0.837	

* Data from 1995 were used for Agadir due to the availability.

**Table A3:** GWR results in the inland cities.

City	Factor	1990		2000		2010		2020	
		Mean	Std.	Mean	Std.	Mean	Std.	Mean	Std.
Fes	Const.	45.224	7.333	39.407	6.42	43.135	6.795	42.079	7.313
	NDVI	−29.072	24.802	19.537	44.358	−14.177	40	24.348	33.829
	NDBI	41.249	21.373	54.638	27.622	49.953	16.672	63.204	17.191
	NDWI	−26.637	19.311	6.267	34.691	−26.88	37.873	1.032	30.39
	DEM	0.001	0.011	0	0.011	−0.003	0.007	0.001	0.009
	Slope	−0.001	0.002	−0.002	0.002	−0.001	0.002	−0.002	0.002
	Aspect	−0.053	0.064	−0.06	0.062	−0.047	0.055	−0.043	0.069
	Hillshade	−0.021	0.014	−0.009	0.013	−0.011	0.014	−0.004	0.018
	Adjusted R^2^	0.781		0.764		0.748		0.831	
Marrakech	Const.	41.763	12.895	52.154	21.946	50.456	19.991	55.711	20.765
	NDVI	−81.418	28.828	−116.335	65.369	−102.411	34.938	−66.571	48.243
	NDBI	12.991	17.18	19.924	19.999	14.043	16.079	25.195	21.608
	NDWI	−55.42	35.825	−103.617	84.045	−90.986	34.512	−63.165	51.512
	DEM	0.004	0.025	−0.026	0.044	−0.01	0.04	−0.015	0.043
	Slope	0	0.001	0	0.001	0	0.001	0.001	0.001
	Aspect	−0.034	0.145	0.02	0.113	0.06	0.178	0.09	0.149
	Hillshade	−0.009	0.026	−0.001	0.031	−0.039	0.041	−0.045	0.034
	Adjusted R^2^	0.795		0.777		0.754		0.693	
Oujda	Const.	51.743	19.628	56.879	19.082	52.265	15.081	52.934	16.561
	NDVI	−36.461	45.437	−32.7	49.317	−19.344	34.913	−1.871	44.051
	NDBI	16.87	24.862	13.893	31.968	24.819	25.435	36.202	28.596
	NDWI	−25.32	59.577	−21.167	52.454	−24.925	34.623	−16.485	34.465
	DEM	−0.006	0.023	−0.01	0.025	−0.003	0.02	−0.005	0.025
	Slope	−0.001	0.002	0	0.001	0	0.001	0	0.001
	Aspect	−0.016	0.144	−0.055	0.134	0.028	0.12	0.007	0.135
	Hillshade	−0.024	0.018	−0.024	0.018	−0.027	0.021	−0.027	0.019
	Adjusted R^2^	0.806		0.846		0.757		0.796	
Laayoune	Const.	33.7	5.481	10.836	29.89	38.069	4.853	36.995	6.818
	NDVI	7.031	28.339	7.615	31.683	−10.605	48.324	25.375	73.329
	NDBI	33.725	18.762	37.698	117.035	24.889	41.982	61.751	59.144
	NDWI	−12.734	41.704	−226.244	218.499	−26.16	58.689	12.622	72.074
	DEM	−0.017	0.024	−0.05	0.115	−0.032	0.037	−0.005	0.035
	Slope	0	0.001	−0.001	0.003	0	0.001	0	0.001
	Aspect	−0.007	0.042	0.078	0.124	−0.012	0.042	0.005	0.032
	Hillshade	−0.004	0.009	−0.001	0.029	−0.001	0.01	−0.001	0.007
	Adjusted R^2^	0.748		0.726		0.821		0.778	
Errachidia	Const.	57.621	22.597	57.528	12.717	73.039	22.098	61.9	14.32
	NDVI	−74.214	80.893	−66.098	54.502	−64.967	72.532	−34.785	36.929
	NDBI	21.335	42.014	12.332	28.008	26.221	37.405	24.489	24.373
	NDWI	−63.112	43.89	−45.168	39.23	−58.741	45.277	−37.562	31.262
	DEM	−0.014	0.02	−0.013	0.011	−0.025	0.018	−0.015	0.012
	Slope	−0.001	0.002	−0.001	0.002	−0.001	0.002	0	0.002
	Aspect	−0.039	0.088	−0.045	0.095	−0.068	0.099	−0.07	0.097
	Hillshade	−0.013	0.02	−0.015	0.013	−0.011	0.017	−0.012	0.019
	Adjusted R^2^	0.910		0.897		0.922		0.903	
